# Decoding the Multifaceted Potential of *Artemisia monosperma*: Comprehensive Insights into Allelopathy, Antimicrobial Activity, and Phytochemical Profile for Sustainable Agriculture

**DOI:** 10.3390/plants12213695

**Published:** 2023-10-26

**Authors:** Mohamed A. El-Sheikh, Anfal Alsharekh, Abdulrahman A. Alatar, Humaira Rizwana

**Affiliations:** Department of Botany & Microbiology, College of Science, King Saud University, P.O. Box 2455, Riyadh 11451, Saudi Arabia; anfal.alsharekh@hotmail.com (A.A.); aalatar@ksu.edu.sa (A.A.A.); hrizwana@ksu.edu.sa (H.R.)

**Keywords:** allelochemicals, antibacterial, antifungal, GC-MS, HPLC, phenolic acids

## Abstract

Weeds present a significant hazard to crop production, necessitating the development of effective and sustainable strategies for weed management. Although synthetic herbicides are effective, concerns about their environmental and health impact have been raised. This study investigates the allelopathic potential, antimicrobial activity, and phytochemical profile of *Artemisia monosperma*. Extracts from *A*. *monosperma* proficiently impede the growth of *Chenopodium murale* and *Amaranthus viridis*, while exhibiting varying effects on crops *Solanum lycopersicum* and *Cucumis sativus*. Leaf and seed extracts demonstrate the most significant inhibition of weed growth. Interestingly, the leaf extract at a concentration of 50% inhibited weed growth in pot experiments without affecting crop growth. Moreover, extracts from *A*. *monosperma* exhibit noteworthy antifungal and antibacterial activity, with the root extract demonstrating the strongest inhibition. The root extract inhibited the mycelial growth of *Colletotrichum musae* by 63% as compared to control. The leaf extract exhibited the highest levels of phenolic acids, in particular gallic acid, amounting to 116.30 ppm. This study emphasizes the multifaceted potential of *A*. *monosperma* as a sustainable solution for weed management and proposes its use in crop protection. Further investigation of its practical applications and optimization of extraction methods can aid in its integration into contemporary agricultural systems, promoting both crop yield and environmental sustainability.

## 1. Introduction

Allelopathy, a fascinating ecological phenomenon, lies at the intersection of plant biology and microorganism interactions. This intricate ecological process occurs when plants and microorganisms release biochemical compounds into their surrounding environment that have either stimulatory or inhibitory effects on the growth, development, or behavior of neighboring organisms. Allelopathy can be broadly defined as “the chemical interference by one organism (either plant or microorganism) with the growth and development of another, mediated through the release of allelochemicals” [1]. These allelochemicals may vary in composition and function, and they can target both species of different taxa, known as interspecies allelopathy, and conspecific individuals within the same species, referred to as intraspecies allelopathy. The study of allelopathy has garnered significant attention in recent years, as it offers valuable insights into ecological interactions, plant–plant competition, and the potential applications of allelopathic compounds in agriculture, weed management, and sustainable crop production [2].

Allelopathy is gaining popularity as a sustainable weed management method for addressing the challenges of environmental pollution and herbicide resistance. This approach aims to use natural plant compounds to suppress weed growth and reduce reliance on chemical herbicides. Its potential for ecological and economic benefits makes it an attractive alternative to traditional weed management strategies. The cultivation of crops, especially in hyper-arid desert regions such as Saudi Arabia, faces numerous challenges during their growth cycles. This is primarily due to the aggressive invasion of these fields by weeds, which take advantage of available niches, moisture, water, nutrients, and shade in these newly acquired habitats. Weeds thus present a formidable challenge to plants during their developmental stages.

Weeds compete with crops for resources, establish themselves rapidly, and cause significant yield losses. These yield losses can be as high as 34% per year and have a significant impact on global crop production [3]. Consequently, weed management has always been a significant hurdle in agricultural fields. Traditional weed management techniques such as polyculture and crop rotation have been considered desirable. However, with the escalating demand for food production, a plethora of methods have been developed, adopted, and implemented. Among these methods, mechanical weeding and herbicides have shown commendable efficacy in recent years [4,5]. While manual and mechanical weeding methods provide favorable results and are generally safe, they are labor-intensive and financially burdensome. Conversely, synthetic herbicides have demonstrated remarkable activity and have been used extensively worldwide to meet the growing demand for crop production [6,7]. However, the excessive use of synthetic chemical herbicides has been associated with adverse effects on both human health and the environment [8,9]. Recent statistics highlight a significant increase in the production and consumption of pesticides on a global scale. In particular, herbicides account for 45% of total expenditure, followed by insecticides at 14% and fungicides at 10%. Herbicides account for the largest share of global consumption in the world market, with a staggering figure of 24,727 million US$ and a steady upward trend [10]. Consequently, there is an urgent need to explore alternative safer, more cost-effective, and more efficient weed management strategies. This requires a comprehensive evaluation of allelopathy as a potential way to harness its beneficial effects while mitigating its negative effects, thereby seeking alternative approaches to weed management.

A novel approach to mitigate the adverse effects of synthetic herbicides in crop production is the use of natural herbicides [11,12,13]. In the complex interplay of crop–weed competition within ecosystems, one of the most influential and often subtle factors is allelopathy [14]. Allelopathy, an important sub-discipline of chemical ecology, is a biological and natural phenomenon. It emphasizes the eco-physiological interactions among higher plants mediated by the secretion of specific chemical compounds known as “allelochemicals”. These allelochemicals are naturally present in various plant components, including roots, seeds, leaves, and stems, in varying concentrations [15]. Most naturally occurring allelochemicals derived from plants exhibit properties that make them non-toxic to humans, environmentally benign (with minimal soil and water contamination), and readily biodegradable [16,17]. They offer the potential to serve as an exceptional, safe, and environmentally friendly weed management strategy. A prominent application of plant allelopathy revolves around the identification of allelochemical activity within phenolic compounds present in plant extracts and their subsequent use as herbicides or for crop protection. Plants or weeds that produce phytotoxic natural products hold great promise for weed management [18]. In particular, certain plant species such as *Acacia melanoxylon* [12], *Conocarpus erectus* [11], *Parthenium hysterophorus* [19], and *Conocarpus* spp. [20] have been documented to produce allelopathic compounds capable of inducing detrimental effects on crops. The existing literature has highlighted that some of these compounds can stimulate crop production and/or inhibit weed growth [11,21,22]. The range of allelochemicals produced by plants is extensive, including chemicals with simple hydrocarbon structures and complex polycyclic aromatics. Nearly every category of secondary metabolites has been linked to allelopathic interactions. For example, phenolic acids, including gallic acid, caffeic acid, and ferulic acid, were proposed as the main contributors to the allelopathic activity of *Conocarpus erectus* leaves [11].

The Asteraceae family is recognized as one of the biggest and most important plant families in the botanical world. Among the members of this family, *Artemisia monosperma* Delile is an exceptional medicinal and aromatic plant frequently found in the Mediterranean region and the Arabian Peninsula. This perennial shrub typically grows 50 to 70 cm tall and thrives in sandy habitats throughout various regions of Saudi Arabia. *A*. *monosperma* has a rich traditional history with documented uses worldwide, including as an antihypertensive, anthelmintic, and antispasmodic agent [23]. *A*. *monosperma* is known to possess elevated concentrations of phenolic compounds and flavonoids, including vanillic acid, ferulic acid, and cinnamic acid, as documented in previous studies [24]. Only one previous study investigated the allelopathic potential of *A*. *monosperma* aqueous extracts against *Medicago polymorpha* L [25]. The results of this study highlighted its robust allelopathic effect, which was particularly evident in germination bioassays.

Harnessing the allelopathic potential of plants to manage weed infestations represents a viable, cost-effective, labor-saving, and environmentally friendly alternative to conventional chemical and mechanical weed control methods. Therefore, the present study was designed to achieve two primary objectives: (1) to evaluate the allelopathic potential of *Artemisia monosperma* against selected weeds, namely *Chenopodium murale* and *Amaranthus viridis*, as well as crops such as *Solanum lycopersicum* and *Cucumis sativus*, by investigating the inhibitory effects of its aqueous extract on their growth, and (2) to identify potential allelochemicals present in this plant in addition to investigate the antimicrobial potential of the studied plant.

## 2. Results

### 2.1. Allelopathic Effect of A. monosperma against Selected Weeds and Crops

#### 2.1.1. *Chenopodium murale*

The findings indicated that extracts from various parts of *A*. *monosperma* effectively impeded the growth of *C*. *murale* seedlings in petri dishes, as revealed in Table 1. Specifically, the leaf extract at 100% concentration resulted in a 93% reduction in root length and an 81% reduction in shoot length. Additionally, the root extract at 100% concentration significantly decreased root length by 63% and shoot length by 47%. Similarly, the seed extract at 100% concentration inhibited 90% of root length and 84% of shoot length. In general, the leaf and seed extracts demonstrated the greatest inhibition, while the root extract produced the least inhibition.

The root lengths of *C*. *murale* were significantly inhibited at the highest concentrations (100%) by the extracts of *A*. *monosperma’s* leaves, roots, and seeds (Table 1). At the 100% concentration, the leaf extract reduced root length by 54% and shoot length by 31%. The root extract also inhibited root length by 55% and shoot length by 30%. Similarly, the seed extract resulted in a 53% decrease in root length and a 27% decrease in shoot length at 100% concentration.

#### 2.1.2. *Amaranthus viridis*

The growth of *A*. *viridis* seedlings grown in petri dishes was significantly inhibited by *A*. *monosperma* extracts, as shown in Table 2. The leaf extract exhibited the highest inhibition of shoot and root lengths, followed by the seed extract, while the root extract recorded the lowest inhibition. At 100% concentration, leaf extract inhibited shoot and root length by 94% and 91%, respectively. At the same concentration, root extract inhibited shoot length by 54% and root length by 51%, while seed extract inhibited shoot and root lengths by 82% each.

*A*. *monosperma* extracts showed a greater inhibitory effect on *A*. *viridis* root length compared to shoot length across all tested concentrations. Generally, root and leaf extracts showed the highest percentage of inhibition on root length growth, followed by the seed extract. At 100% concentration, leaf, root, and seed extracts inhibited root length growth by 61%, 71%, and 47%, respectively (Table 2). In comparison, shoot length inhibition was observed to be 16%, 29%, and 23% by leaf, root, and seed extracts, respectively.

#### 2.1.3. *Solanum lycopersicum*

*A*. *monosperma* root extract promoted the growth of *S*. *lycopersicum* seedlings, as demonstrated in petri dish experiments (Table 3). The root extract increased shoot lengths at concentrations of 25%, 50%, and 75%, but decreased shoot length by 1% at a concentration of 100%. Conversely, the leaf extract inhibited root and shoot growth by 95% and 78%, respectively, while the seed extract resulted in a 86% and 77% reduction in root and shoot lengths at a concentration of 100%. Our results indicated that the leaf extract of *A*. *monosperma* showed the highest inhibition, followed by the seed extract, with the root extract exhibiting the lowest inhibition.

The data in Table 3 demonstrate that leaf, root, and seed extracts did not effectively inhibit the growth of root and shoot lengths in *S*. *lycopersicum* during the pot experiments. Specifically, the 100% concentration of leaf extract resulted in a 9% and 34% reduction in root and shoot lengths, respectively. Root extracts at 100% concentration led to inhibition of 26% and 3% in root and shoot lengths, respectively. Similarly, the seed extract decreased root and shoot lengths by 18% and 22%, respectively, at 100% concentration. However, the growth inhibition of both root and shoot lengths was not significant in pot experiments.

#### 2.1.4. *Cucumis sativus*

It was observed that the seedling growth of *C*. *sativus* was stimulated in experiments using 25% and 50% concentrations of root and seed extracts of *A*. *monosperma* (Table 4). However, at 100% concentration, significant inhibition of root and shoot growth was observed in the leaf and root extracts. At 100%, the leaf extract inhibited root and shoot lengths by 99% each, while the root extract inhibited root and shoot lengths by 99% and 98%, respectively. Moreover, the seed extract decreased root and shoot length by 66% and 55%, respectively, at a concentration of 100%. Overall, the leaf and root extracts demonstrated the greatest inhibition, while the seed extract had the least impact.

In pots, *A*. *monosperma* extracts promoted the growth of *C*. *sativus* seedlings. At a 50% concentration, leaf extract stimulated the lengths of both shoots and roots. Root extract promoted shoot length at 25% and 100% concentrations while seed extract stimulated shoot length at 25% and 50% concentration. No significant inhibition percentages were observed for any of the extracts. Leaf and seed extracts caused a 13% and 7% reduction in root length at 100% concentration, respectively, while no reduction was observed with root extract.

### 2.2. Antimicrobial Potential of A. monosperma

#### 2.2.1. Antifungal Effect

**Figure 1:**
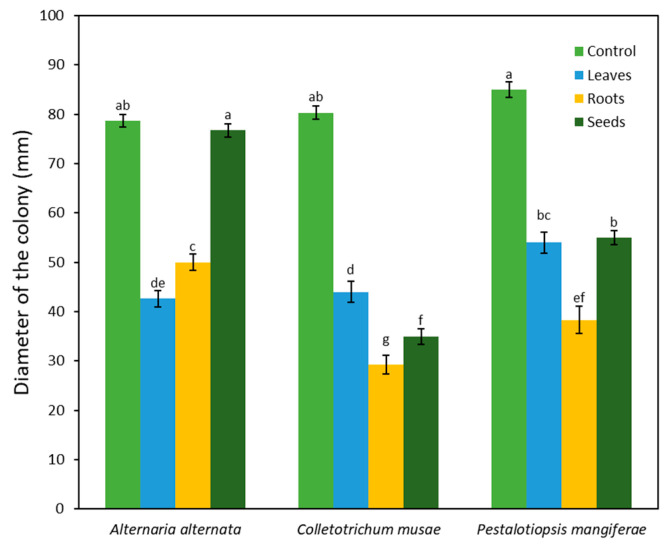
The antifungal potential of *Artemisia monosperma*. The leaf, root, and seed extracts showed significant antifungal activity against three selected fungal species showed in terms of radial mycelial growth. Each column represents the mean of 5 replicates. Error bars show the standard deviation of their respective mean. Columns with the same letter are not significantly different (*p* ≤ 0.05).

Extracts from the leaves, roots, and seeds of *A*. *monosperma* were examined for their ability to inhibit three phytopathogenic fungi. The results demonstrated that all the examined parts were capable of significantly inhibiting *Colletotrichum musae* (Figure 1). The root extract exhibited the highest level of mycelial growth inhibition at 63%, followed by the seed and leaf extracts at 56% and 45%, respectively. The mycelial growth of *Alternaria alternata* was inhibited by 46% and 36% through extracts from the roots and leaves, while the seed extract only showed a negligible 3% inhibition. In a similar manner, all plant parts inhibited *Pestalotiopsis mangiferae*, with the root extract causing the greatest mycelial growth inhibition at 55%, followed by the leaf and seed extracts at 36% and 35%, respectively. Root extract proved most effective in inhibiting all fungal isolates in contrast to leaf and seed extracts.

#### 2.2.2. Antibacterial Activity

**Figure 2:**
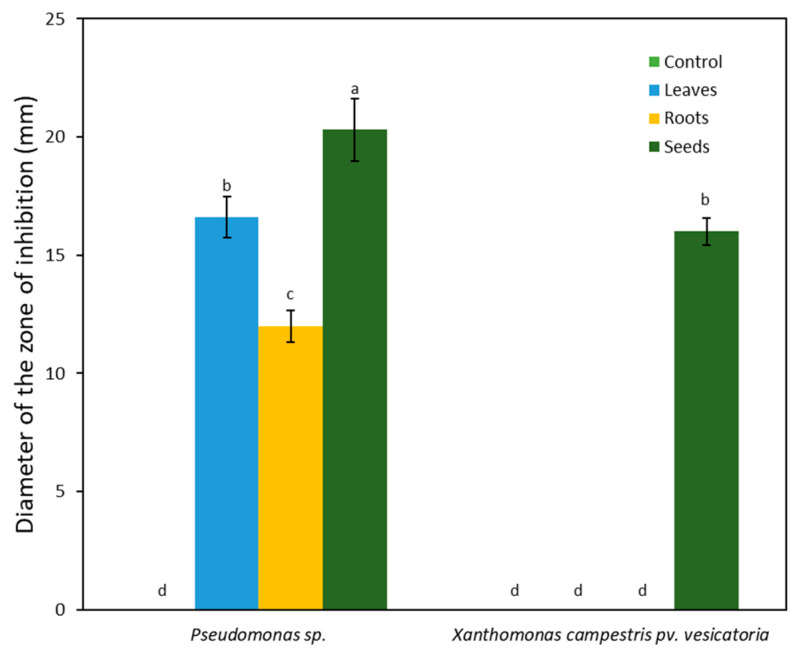
The antibacterial potential of *Artemisia monosperma*. Seed extract showed strong antibacterial inhibition activity against the two selected bacterial strains, while the antibacterial effects of leaf and root extracts were variable. Each column represents the mean of 5 replicates. Error bars shows the standard deviation of their respective mean. Columns with the same letter are not significantly different (*p* ≤ 0.05).

Extracts from the leaves, roots, and seeds of *A*. *monosperma* significantly inhibited the growth of *Pseudomonas* sp., while inhibition against *Xanthomonas campestris* pv. *vesicatoria* was minimal. The mean diameters of the inhibition zones for *Pseudomonas* sp. by leaf, root, and seed extracts were 16.60, 12.00, and 20.30 mm, respectively (Figure 2). *Xanthomonas campestris* pv. *vesicatoria* was not inhibited by the root and leaf extracts, but treatment with seed extract resulted in an inhibition zone of 16.00 mm.

### 2.3. Chemical Profiling of A. monosperma

#### 2.3.1. Quantification of Phenolic Acids by HPLC

The extract of various parts (i.e., leaves, roots, and seeds) of *A*. *monosperma* underwent HPLC with authentic references to quantify the phenolic contents (i.e., gallic, caffeic, and ferulic acids). The leaf extract displayed a high level of gallic acid at 116.30 ppm, while ferulic acid was found to be the lowest at 9.06 ppm (Table 5). The root extract contained 12.82, 10.59, and 7.56 ppm of gallic acid, caffeic acid, and ferulic acids, respectively. In seed extract, ferulic acid had the highest concentration of at 63.10 ppm, followed by gallic acid at 17.60 ppm and caffeic acid at 10.56 ppm.

#### 2.3.2. Phytochemical Screening Using GC-MS

**Figure 3:**
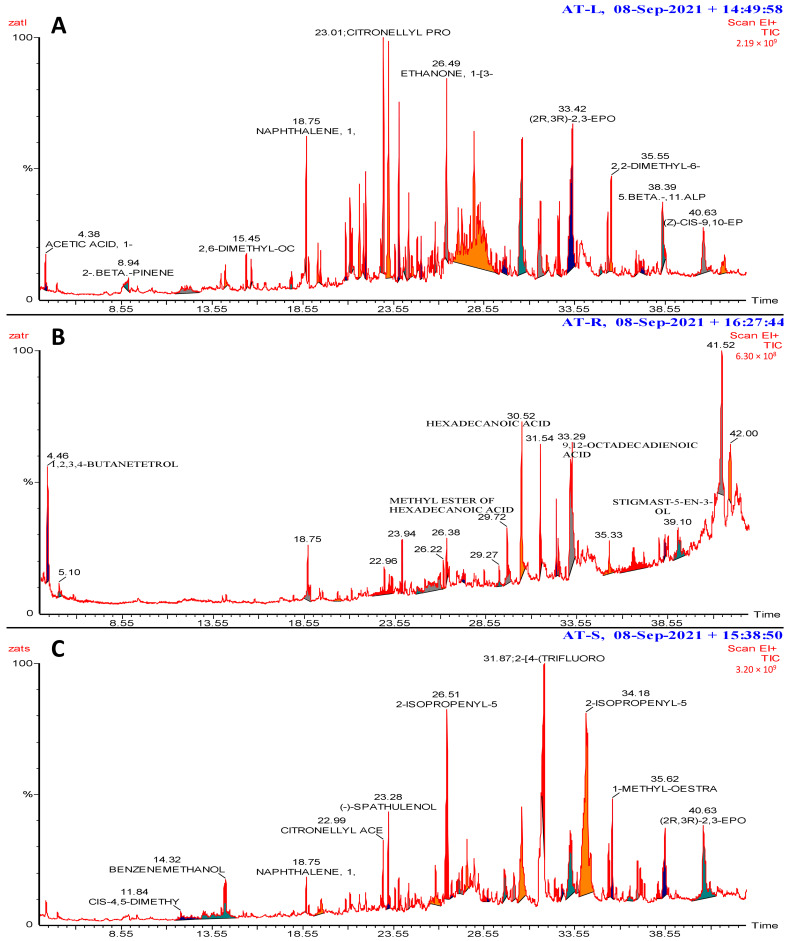
GC-MS spectrum showing some of the identified compounds in the extracts of leaves (**A**), roots (**B**), and seeds (**C**) of *Artemisia monosperma*. The full list of the identified compounds in each extract along with their retention time and area values is in the Supplementary Table S1.

GC-MS analyses were performed on the leaf, root, and seed extracts of *A*. *monosperma*. The results revealed that the leaf extract contained thirty-three phytochemical compounds (Supplementary Table S1). Notable compounds included 2,6-dimethyl-octa-2,6-dien-1-ol, citronellyl propanoate, and ethenone (Figure 3A). In the root extract, prominent peaks were identified including Butane-1,2,3,4-tetrol, Lavandulyl acetate, Epoxypinane, trans-(-)-gamma-irone, 2, 6, 10-Cycloundecatrien-1-one, 2,5-Diacetylanisole, Hexadecanoic acid methyl ester, and 9,12-Octadecadienoic acid (Figure 3B). The seed extract showed eighteen compounds (Supplementary Table S1). Some of the significant compounds detected were 2,5-dimethyl-4-octene, Citronellol acetate, Spathulenol, and 2-Isopropenyl-5-acetyl-2,3-dihydrobenzofuran (Figure 3C).

## 3. Discussion

Allelopathy in agroecosystems can have both beneficial and harmful effects on target plants, soil microbiota, and the broader environment. Depending on the allelochemicals in donor plants, it has the potential to improve agricultural productivity by suppressing weed growth and protecting crops from diseases. However, it may also cause autotoxicity and soil deterioration, leading to negative consequences [1,26]. The phytotoxic effects of phenolic compounds in plant extracts are a recognized strategy for weed control and crop protection [15,19,27,28]. In light of this investigation, it is clear that leaf extracts from *A*. *monosperma* plants contain a significant content of phenolic compounds, indicating their potential for allelopathic effects against weeds. The results of the current study indicate that the toxicity of phenolic compounds in *A. monosperma* extracts varies based on the plant parts and their concentrations, which is consistent with previous research [11,20]. Additionally, the allelopathic effects of *A*. *monosperma* phenolic compounds differ when acting on weeds (*C*. *murale*, *A*. *viridius*) versus crops (*S*. *lycopersicum*, *C*. *sativus*). Notably, the phenolic compounds identified in various parts of *A*. *monosperma*, including gallic acid, ferulic acid, and caffeic acid, are acknowledged as environmentally friendly and naturally occurring [20,29].

*A*. *monosperma* demonstrated significant antimicrobial activity against bacteria and fungi in our study, highlighting the inhibitory potential of its secondary metabolites with noteworthy biological properties. Notably, the extracts of the plant exhibited a significant inhibitory effect on *Pestalotiopsis mangiferae*, with an inhibition rate of 55%. While the effects on bacterial isolates from other tested plants were relatively weaker, our findings are consistent with prior research which has highlighted the potent antibacterial and antifungal activity of *A*. *monosperma* [30,31].

Overall, the allelopathic activity of *A*. *monosperma* extracts showed noteworthy inhibitory effects on the growth of weed seedlings in both Petri dishes and pots. Notably, the leaves of *A*. *monosperma* proved to be the most potent plant part in inhibiting the growth of both weed species, specifically *C*. *murale* and *A*. *viridis*. Previous research has shown that phenolic compounds, including gallic acid, caffeic acid, and ferulic acid, can hinder weed growth by inducing physiological changes, such as water stress, suppression of photosynthetic rates, and interference with enzyme function [32,33]. Interestingly, the bioassay results displayed higher inhibition rates than those observed in soil-based experiments. This difference could be due to the solubility of certain phenolic compounds in water, which can seep from the roots of the plants under scrutiny and into the soil, possibly decreasing their ability to inhibit growth [34].

In contrast to weeds, both tomato and cucumber plants showed resistance to the allelopathic effects of *A*. *monosperma* extracts when cultivated in either Petri dishes or pots. Tomato plants exhibited minimal to no inhibition, except under high concentrations of leaf extracts in Petri dishes. Similarly, cucumber growth was only affected by extracts at high concentrations in Petri dishes. This resistance of tomato and cucumber to phenolic compounds can be attributed to their limited susceptibility. Weed seeds, smaller in size than crop seeds, are more vulnerable to allelochemicals due to their lower carbohydrate storage capacity [35]. Previous studies have suggested that small-seeded weeds can be effectively controlled through allelopathic activity, whereas larger-seeded crops like *C*. *sativus* may not be as affected [36]. Additionally, the examined weeds may be more sensitive to phenolic acids than the studied crops, namely tomato and cucumber. These experiments highlight the importance of extract concentration and source in determining phytotoxicity, showing potential for the creation of environmentally friendly herbicides for agricultural use.

## 4. Materials and Methods

### 4.1. Collection of Plant Materials

Leaves, roots, and seeds of the donor plant *Artemisia monosperma*, as well as seeds of target weeds *Chenopodium murale* and *Amaranthus viridis*, were locally sourced from various regions in Riyadh, Saudi Arabia, between 2019 and 2020. Seeds of target crops, including *Solanum lycopersicum* (AC 55 VF, Pomodoro, League City, TX, USA) and *Cucumis sativus* (Beta Alpha, Agrimaxspin, Dallas Seeds, Dallas, TX, USA), were obtained from a commercial seed supplier. The seeds of targeted crops and weeds were sterilized by soaking them in a 70% ethanol solution for 2 min, followed by a 5 min treatment with 2.0% sodium hypochlorite (NaOCl). The sterilized seeds were then rinsed five times with sterile distilled water.

### 4.2. Preparation of Aqueous Extracts

Leaves, roots, and seeds of the donor plant (*A*. *monosperma*) were harvested from three separate plants and extracted individually. The harvested plant parts were thoroughly washed with running water and then rinsed with sterile distilled water before being air-dried in the shade for 2 to 3 weeks at room temperature. The dried plant parts were then finely ground into a powder. Various aqueous extracts were prepared by immersing 1 g of dried plant powder from the respective plant parts into 100 mL of sterile distilled water for 24 h on a shaker set at 180 rpm. The extracts were filtered using cheesecloth, followed by filtration through Whatman filter paper No. 1, as described by Hussain, El-Sheikh and Reigosa [12]. The resulting filtrate was considered a 100% solution. Dilutions were made to generate varying concentrations (75%, 50%, and 25%). These reconstituted extracts were used in subsequent bioassays and growth experiments.

### 4.3. Petri-Dish Bioassay of Seed Germination

In Petri dishes with a double layer of sterile filter paper, five seeds from the target plants were placed in three replicates. Following this, 5 mL of donor plant extracts (leaves, roots, or seeds) were added to the Petri dishes for each concentration. The control group for each target plant was treated with distilled water only. The Petri dishes were positioned under cool fluorescent light with an intensity of 350 µmol m^−2^ s^−1^ at a temperature of 25 °C, adhering to a 12 h light and 12 h dark photoperiod. Seedling and radical growth of the recipient plants were then assessed using a ruler following a treatment period of 7 to 14 days. Each treatment was replicated five times, and the experimental design employed was a completely randomized design (CRD).

### 4.4. Growth Inhibition by Aqueous Extracts

Aqueous extracts from the donor plant were combined with sterilized potting soil in individual plastic pots measuring 30 cm in diameter (pH range 5.0–6.0, Bass Van Buuren, Maasland, The Netherlands) at varying concentrations of 100%, 75%, 50%, and 25% *v*/*v*. Subsequently, seeds of the target plants were sown in these pots. Each treatment was repeated five times, with each of the five replicates featuring three pots containing five target plant seeds. Sterilized distilled water was administered to the pots every other day for a period ranging from 7 to 14 days. Following this, measurements were taken of the root and shoot lengths. This study followed a randomized complete block design (RCBD).

### 4.5. Antimicrobial Potential of Artemisia Monosperma

The antimicrobial activities of various extracts were assessed against plant pathogenic fungi and bacteria. The phytopathogens selected for this study were isolated from infected crop plants cultivated in agricultural regions of Saudi Arabia. The tested fungi included *Alternaria alternata*, *Colletotrichum musae*, and *Pestalotiopsis mangiferae*, while the bacterial strains included *Pseudomonas* sp. and *Xanthomonas campestris* pv. *vesicatoria*.

#### 4.5.1. Antifungal Effects

One milliliter of the extract was dispensed onto sterile Petri dishes, and fifteen milliliters of potato dextrose agar (PDA) were then added. After gently swirling the mixture to ensure thorough mixing, it was allowed to cool and solidify. Subsequently, a 6 mm mycelial disc obtained from the periphery of a seven-day-old fungal colony from actively growing fungal culture plates was centrally inoculated onto each dish. All tested fungi underwent the identical inoculation procedure and the inoculated plates were incubated at a controlled temperature of 25 °C ± 1 °C for 7 days. The control treatment did not receive any extract application. The extract’s antifungal activity was evaluated by measuring mycelial growth after the 7-day incubation period. Each experiment was carried out in five replicates. Mycelial plugs displaying no visible fungal growth were transferred to fresh PDA medium to verify the viability of each fungus on the plug.

The percentage of mycelial growth inhibition was determined [37]:

(1) $$I=\frac{C-T}{C}\times 100$$

where: *I*: Percentage inhibition, *T*: Average mycelial growth after treatment, *C*: Average mycelial growth in the control plate (untreated).

#### 4.5.2. Antibacterial Effects

Antibacterial activity was assessed using the well diffusion method [38]. Sterile Petri dishes were filled with 15 mL of agar Mueller–Hilton (MH) and left to solidify. To prepare the bacterial suspension, a nutrient broth was cultured overnight and adjusted to a concentration of 10^7^ colony-forming units (CFU), as compared to the McFarland turbidity standard. The suspension was evenly spread onto the agar MH plates. Following this step, wells were created in the agar HM using a cork borer. Each well was loaded with 200 µL of different extract concentrations individually. The control treatment remained without any extract application. The plates were incubated at 35 °C for 24 h. Each experiment was conducted in five replicates. The presence of inhibition zones indicated antibacterial activity, and the zones were measured with the measurements accurately recorded.

### 4.6. Phytochemical Profiling via GC-MS

The analysis using gas chromatography–mass spectrometry (GC-MS) was performed at the Research Center of the College of Pharmacy, King Saud University, Riyadh, by utilizing a Perkin Elmer Clarus 600 T gas chromatograph coupled with a Turbomass spectrometer, following the method previously reported by Nasr, et al. [39]. To each extract, a 1 µL aliquot was injected into an Elite-5MS column, which measures 30 m in length, has a 0.25 µm film thickness, and an internal diameter of 0.25 mm. The GC-MS system was programmed to start with an initial oven temperature of 40 °C, held for 2 min, followed by an incremental increase to 150 °C at a rate of 5 °C every 2 min, and finally increased to 300 °C at a rate of 5 °C per 5 min. The injector temperature was consistently maintained at 280 °C, while the interface temperature was set at 250 °C. The mobile phase used was helium, with a flow rate of 1.0 mL min^−1^. Mass spectral detection was performed in electron ionization mode, scanning over a range of 40 to 600 (m z^−1^). To identify unknown compounds, we compared their spectral profiles with the National Institute of Standards and Technology library. Additionally, we confirmed the identities of phytochemical compounds by considering factors such as peak area, retention time, and molecular formula.

### 4.7. Phenolic Acids Quantification via HPLC

Phenolic acids were quantified through high-performance liquid chromatography (HPLC) with UV detection on the Alliance 2695 Separations Module (Waters Instruments, Inc. in Milford, MA, USA). The analyses were performed following the method of Wen, et al. [40] with slight modifications utilizing a Shimadzu Pinnacle C18 column, 250 × 4.6 mm, 5 μm, and a reverse-phase technique. The mobile phase was composed of (A) 2% acetic acid in acidified water and (B) acetonitrile and methanol in a 65:35 (*v*/*v*) ratio, with a flow rate of 1 mL min^−1^. The optimized gradient program followed this sequence: 0–10 min (10–45% B), 10–20 min (45–90% B), 20–23 min (90–10% B), and 23–25 min (10% B). Samples were injected at 10 µL and analyzed at a single wavelength of 280 nm.

### 4.8. Statistical Analysis

All the data gathered underwent analysis using the Statistical Package for the Social Sciences (SPSS^®^) Statistics 28 (IBM, Armonk, NY, USA). Two-way analysis of variance (ANOVA) was utilized with the donor plant part and solution concentration serving as the two independent factors. Duncan’s Multiple Range Test (DMRT) was used to compare means, with a significance level of 0.05.

## 5. Conclusions

The study found that extracts from *Artemisia monosperma* significantly inhibited weed growth, while having minimal to non-impact on the growth of crops such as tomatoes and cucumbers. Notably, extracts from the leaves of *A*. *monosperma* had the greatest inhibitory effect on weed growth at varying concentration levels. These findings provide a crucial basis for future research exploring the possibility of using *A*. *monosperma* extracts for biological weed control via allelopathic mechanisms. It is recommended that additional studies be conducted to determine the feasibility of applying these extracts on a large scale and to evaluate their potential impact on crop growth and overall agricultural output.

## Figures and Tables

**Table 1:** The impact of *Artemisia monosperma* leaf, root, and seed extracts at various concentrations (0%, 25%, 50%, 75%, and 100%) on the seed germination and growth of *Chenopodium murale* seedlings.

Plant Parts	Concentration (%)	Seed Germination		Plant Growth	
		Shoot Length (cm)	Root Length (cm)	Shoot Length (cm)	Root Length (cm)
Leaves *	0	5.26 ± 0.17 ^a^	3.19 ± 0.32 ^a^	5.40 ± 0.21 ^a^	2.85 ± 0.41 ^a^
	25	2.65 ± 0.08 ^bc^	1.10 ± 0.07 ^ce^	3.92 ± 0.19 ^b^	1.83 ± 0.25 ^ab^
	50	2.17 ± 0.05 ^cd^	0.82 ± 0.13 ^ce^	4.00 ± 0.16 ^b^	1.45 ± 0.14 ^b^
	75	1.61 ± 0.18 ^de^	0.24 ± 0.03 ^e^	4.03 ± 0.21 ^b^	1.55 ± 0.14 ^b^
	100	1.00 ± 0.17 ^ef^	0.23 ± 0.04 ^e^	3.70 ± 0.45 ^b^	1.31 ± 0.16 ^b^
Roots	0	5.26 ± 0.17 ^a^	3.19 ± 0.31 ^a^	5.40 ± 0.21 ^a^	2.85 ± 0.41 ^a^
	25	3.20 ± 0.11 ^b^	3.07 ± 0.24 ^a^	4.41 ± 0.27 ^ab^	1.87 ± 0.12 ^ab^
	50	3.21 ± 0.14 ^b^	2.38 ± 0.21 ^ab^	4.2 ± 0.14 ^b^	2.35 ± 0.17 ^ab^
	75	3.15 ± 0.12 ^b^	1.50 ± 0.18 ^bc^	4.23 ± 0.21 ^ab^	1.48 ± 0.21 ^b^
	100	2.79 ± 0.15 ^bc^	1.19 ± 0.14 ^cd^	3.80 ± 0.19 ^b^	1.28 ± 0.14 ^b^
Seeds	0	5.26 ± 0.17 ^a^	3.19 ± 0.34 ^a^	5.40 ± 0.21 ^a^	2.85 ± 0.41 ^a^
	25	2.61 ± 0.15 ^bc^	0.98 ± 0.11 ^ce^	4.55 ± 0.14 ^ab^	1.72 ± 0.09 ^b^
	50	2.34 ± 0.08 ^c^	0.67 ± 0.06 ^ce^	4.71 ± 0.12 ^ab^	1.81 ± 0.14 ^ab^
	75	1.66 ± 0.14 ^de^	0.34 ± 0.06 ^de^	4.57 ± 0.11 ^ab^	1.86 ± 0.09 ^ab^
	100	0.85 ± 0.09 ^f^	0.33 ± 0.05 ^de^	3.93 ± 0.47 ^b^	1.34 ± 0.16 ^b^
*f*-values	Part	85.27	67.38	3.81	0.70
	Concentration	323.08	100.15	17.71	18.86
	Part × Concentration	9.91	6.11	0.46	1.02
*p*-values	Part	0.00	0.00	0.02	0.50
	Concentration	0.00	0.00	0.00	0.00
	Part × Concentration	0.00	0.00	0.88	0.43

* Values are shown as mean of 5 replicates ± standard deviation. Values in the same column followed by the same letter are not significantly different (*p* ≤ 0.05).

**Table 2:** *Artemisia monosperma* extracts’ effects on *Amaranthus viridis’* seed germination and plant growth. The extracts were derived from leaves, roots, and seeds, and tested at varying concentrations (0%, 25%, 50%, 75%, and 100%).

Plant Parts	Concentration (%)	Seed Germination		Plant Growth	
		Shoot Length (cm)	Root Length (cm)	Shoot Length (cm)	Root Length (cm)
Leaves *	0	2.26 ± 0.07 ^ab^	1.48 ± 0.07 ^a^	4.05 ± 0.17 ^a^	1.90 ± 0.10 ^a^
	25	1.91 ± 0.15 ^ab^	0.81 ± 0.06 ^bc^	3.03 ± 0.23 ^ab^	0.75 ± 0.10 ^bd^
	50	1.73 ± 0.13 ^bc^	0.55 ± 0.06 ^ce^	2.4 ± 0.44 ^b^	0.54 ± 0.12 ^d^
	75	0.64 ± 0.17 ^ef^	0.33 ± 0.09 ^ef^	2.98 ± 0.37 ^ab^	0.66 ± 0.12 ^cd^
	100	0.21 ± 0.11 ^f^	0.09 ± 0.05 ^f^	3.40 ± 0.44 ^ab^	0.75 ± 0.10 ^bd^
Roots	0	2.26 ± 0.07 ^ab^	1.48 ± 0.07 ^a^	4.05 ± 0.17 ^a^	1.9 ± 0.10 ^a^
	25	2.35 ± 0.08 ^a^	1.69 ± 0.04 ^a^	3.69 ± 0.28 ^ab^	0.79 ± 0.17 ^bd^
	50	1.88 ± 0.07 ^ab^	1.06 ± 0.06 ^b^	3.45 ± 0.27 ^ab^	0.79 ± 0.18 ^bd^
	75	1.28 ± 0.16 ^cd^	0.80 ± 0.10 ^bc^	3.39 ± 0.31 ^ab^	0.52 ± 0.15 ^d^
	100	1.05 ± 0.24 ^de^	0.72 ± 0.68	2.86 ± 0.53 ^ab^	0.55 ± 0.15 ^d^
Seeds	0	2.26 ± 0.07 ^ab^	1.48 ± 0.07 ^a^	4.05 ± 0.17 ^a^	1.9 ± 0.10 ^a^
	25	1.15 ± 0.08 ^de^	0.97 ± 0.07 ^b^	3.83 ± 0.23 ^ab^	1.28 ± 0.10 ^b^
	50	0.73 ± 0.08 ^df^	0.51 ± 0.05 ^ce^	3.84 ± 0.30 ^ab^	1.23 ± 0.08 ^bc^
	75	0.61 ± 0.04 ^ef^	0.35 ± 0.03 ^def^	3.22 ± 0.26 ^ab^	1.18 ± 0.08 ^bc^
	100	0.41 ± 0.05 ^f^	0.27 ± 0.04 ^ef^	3.13 ± 0.364 ^ab^	1.00 ± 0.15 ^bd^
*f*-values	Part	48.39	61.76	2.54	17.60
	Concentration	104.21	111.20	4.24	44.99
	Part × Concentration	7.61	5.15	1.46	1.79
*p*-values	Part	0.00	0.00	0.08	0.00
	Concentration	0.00	0.00	0.00	0.00
	Part × Concentration	0.00	0.00	0.18	0.08

* Values are shown as mean of 5 replicates ± standard deviation. Values in the same column followed by the same letter are not significantly different (*p* ≤ 0.05).

**Table 3:** The effect of leaf, root, and seed extracts from *Artemisia monosperma* using varying concentrations (0%, 25%, 50%, 75%, and 100%) on the seed germination and growth of *Solanum lycopersicum* seedlings.

Plant Parts	Concentration (%)	Seed Germination		Plant Growth	
		Shoot Length (cm)	Root Length (cm)	Shoot Length (cm)	Root Length (cm)
Leaves *	0	6.94 ± 0.26 ^a^	6.10 ± 0.32 ^a^	9.43 ± 0.23 ^ab^	3.24 ± 0.24 ^ab^
	25	4.78 ± 0.17 ^b^	1.16 ± 0.16 ^dg^	8.4 ± 0.40 ^bc^	2.99 ± 0.24 ^ab^
	50	3.57 ± 0.22 ^bc^	1.16 ± 0.18 ^dg^	7.52 ± 0.48 ^cd^	2.59 ± 0.19 ^ab^
	75	3.17 ± 0.19 ^cd^	0.91 ± 0.11 ^efg^	7.82 ± 0.57 ^bd^	2.63 ± 0.24 ^ab^
	100	1.47 ± 0.21 ^e^	0.28 ± 0.05 ^g^	6.24 ± 0.54 ^d^	2.95 ± 0.27 ^ab^
Roots	0	6.94 ± 0.26 ^a^	6.1 ± 0.32 ^a^	9.43 ± 0.23 ^ab^	3.24 ± 0.24 ^ab^
	25	7.45 ± 0.30 ^a^	5.31 ± 0.38 ^ab^	9.19 ± 0.24 ^abc^	2.63 ± 0.19 ^ab^
	50	7.41 ± 0.35 ^a^	4.78 ± 0.31 ^b^	9.16 ± 0.39 ^abc^	2.94 ± 0.23 ^ab^
	75	7.40 ± 0.27 ^a^	4.13 ± 0.44 ^b^	9.06 ± 0.37 ^abc^	2.40 ± 0.36 ^ab^
	100	6.84 ± 0.36 ^a^	2.63 ± 0.31 ^c^	10.51 ± 0.27 ^a^	2.78 ± 0.21 ^ab^
Seeds	0	6.94 ± 0.26 ^a^	6.1 ± 0.32 ^a^	9.43 ± 0.23 ^ab^	3.24 ± 0.24 ^ab^
	25	3.87 ± 0.44 ^bc^	2.04 ± 0.17 ^cdf^	8.11 ± 0.38 ^bd^	2.40 ± 0.52 ^ab^
	50	1.93 ± 0.11 ^de^	2.22 ± 0.32 ^cde^	8.19 ± 0.23 ^bc^	3.26 ± 0.94 ^ab^
	75	1.92 ± 0.16 ^de^	2.43 ± 0.15 ^cd^	9.41 ± 0.48 ^ab^	3.15 ± 0.67 ^ab^
	100	1.58 ± 0.26 ^e^	0.88 ± 0.18 ^fg^	7.31 ± 0.48 ^cd^	2.64 ± 0.75 ^ab^
*f*-values	Part	308.14	127.36	21.59	0.43
	Concentration	81.43	132.26	5.79	2.66
	Part × Concentration	23.86	10.16	5.75	1.49
*p*-values	Part	0.00	0.00	0.00	0.65
	Concentration	0.00	0.00	0.00	0.04
	Part × Concentration	0.00	0.00	0.00	0.16

* Values are shown as mean of 5 replicates ± standard deviation. Values in the same column followed by the same letter are not significantly different (*p* ≤ 0.05).

**Table 4:** Effect of *Artemisia monosperma* extracts at varying concentrations (0%, 25%, 50%, 75%, 100%) on seedling germination and growth of *Cucumis sativus*.

Plant Parts	Concentration (%)	Seed Germination		Plant Growth	
		Shoot Length (cm)	Root Length (cm)	Shoot Length (cm)	Root Length (cm)
Leaves *	0	7.09 ± 0.26 ^ac^	10.22 ± 0.77 ^bc^	12.51 ± 0.37 ^bcd^	11.20 ± 0.47 ^ab^
	25	1.94 ± 0.63 ^ef^	1.35 ± 0.47 ^d^	11.95 ± 0.84 ^cd^	10.15 ± 0.72 ^ab^
	50	0.27 ± 0.23 ^f^	0.19 ± 0.17 ^d^	12.70 ± 0.42 ^bcd^	12.75 ± 0.98 ^a^
	75	0.22 ± 0.20 ^f^	0.15 ± 0.13 ^d^	10.86 ± 0.61 ^d^	12.00 ± 1.08 ^a^
	100	0.03 ± 0.019 ^f^	0.02 ± 0.01 ^d^	11.04 ± 0.46 ^d^	11.24 ± 0.95 ^ab^
Roots	0	7.09 ± 0.26 ^ac^	10.22 ± 0.77 ^bc^	12.51 ± 0.37 ^bcd^	11.20 ± 0.47 ^ab^
	25	8.50 ± 0.96 ^a^	11.56 ± 1.66 ^ac^	15.70 ± 0.198 ^a^	9.70 ± 0.72 ^ab^
	50	8.69 ± 1.08 ^a^	12.68 ± 1.20 ^ab^	14.55 ± 0.39 ^ab^	8.00 ± 1.28 ^b^
	75	0.13 ± 0.03 ^f^	0.08 ± 0.02 ^d^	11.58 ± 0.67 ^d^	7.96 ± 0.67 ^b^
	100	0.09 ± 0.03 ^f^	0.05 ± 0.01 ^d^	14.13 ± 0.99 ^ac^	11.27 ± 0.59 ^ab^
Seeds	0	7.09 ± 0.26 ^ac^	10.22 ± 0.77 ^bc^	12.51 ± 0.37 ^bcd^	11.20 ± 0.47 ^ab^
	25	7.91 ± 0.291 ^ab^	15.17 ± 0.96 ^a^	12.58 ± 0.32 ^bcd^	10.40 ± 0.48 ^ab^
	50	5.94 ± 0.46 ^bc^	14.01 ± 0.87 ^ab^	12.82 ± 0.36 ^bcd^	10.55 ± 0.46 ^ab^
	75	4.88 ± 0.72 ^cd^	8.08 ± 1.46 ^c^	12.47 ± 0.35 ^bcd^	10.94 ± 0.67 ^ab^
	100	2.99 ± 0.47 ^de^	3.47 ± 0.69 ^d^	12.19 ± 0.33 ^bcd^	10.39 ± 0.40 ^ab^
*f*-values	Part	80.35	109.01	17.10	7.75
	Concentration	84.47	74.88	6.12	1.19
	Part × Concentration	21.04	20.52	3.47	2.78
*p*-values	Part	0.00	0.00	0.00	0.00
	Concentration	0.00	0.00	0.00	0.32
	Part × Concentration	0.00	0.00	0.00	0.01

* Values are shown as mean of 5 replicates ± standard deviation. Values in the same column followed by the same letter are not significantly different (*p* ≤ 0.05).

**Table 5:** Contents of phenolic compounds (ppm) in leaf, root, and seed extracts of *Artemisia monosperma* as revealed by HPLC analysis.

Phenolic Acid Compounds (ppm)	Leaves	Roots	Seeds
Gallic acid *	18.60 ± 0.20 ^c^	12.82 ± 0.40 ^d^	17.60 ± 0.70 ^c^
Caffeic acid	116.30± 0.30 ^a^	10.59 ± 0.29 ^e^	10.56 ± 0.43 ^e^
Ferulic acid	9.06 ± 0.16 ^ef^	7.56 ± 0.29 ^f^	63.10 ± 0.58 ^b^

* Values are shown as mean of 5 replicates ± standard deviation. Values followed by the same letter are not significantly different (*p* ≤ 0.05).

## Data Availability

The data presented in this study are available on request from the corresponding author.

## Supplementary Materials

The following supporting information can be downloaded at: https://www.mdpi.com/article/10.3390/plants12213695/s1, Table S1: Phytochemical composition of *Artemisia monosperma* as revealed by the GC-MS analysis.
